# The Combination of Zerumbone with 5-Fluorouracil for Sensitizing Colorectal Cancer-Associated Fibroblasts to Treatment

**DOI:** 10.1155/2022/9369328

**Published:** 2022-04-11

**Authors:** Sima Nobari, Rezvan Najafi, Ali Mahdavinezhad, Akram jalali, Razieh Amini

**Affiliations:** Research Center for Molecular Medicine, School of Medicine, Hamadan University of Medical Sciences, Hamadan, Iran

## Abstract

The present study aimed to evaluate the synergic effects of combination therapy on 5-fluorouracil (5-FU) resistance-cancer-associated fibroblasts (CAFs) to treatment. Chemotherapy resistance is an important challenge in colorectal cancer (CRC) eradication attention to the tumor microenvironment (TME) is very important. CAFs in the TME play an essential role in cancer chemoresistance and relapse. Additionally, many patients with advanced CRC show resistance to 5-FU therapy. Anti-tumorigenic activities of ZER, a chemopreventive compound derived from the rhizomes of the wild ginger, have been demonstrated. Synergistic and potentiating effects of combination therapy, using herbal and chemical drugs, can improve patients' response. At the first, CAFs were isolated from a CRC patient and sorted by fluorescent-activated cell sorting (FACS), then, confirmed by flow cytometry, and immunocytochemistry (ICC). The effect of 5-FU and ZER on the cell viability was investigated by MTT assay in a dose and time-dependent manner, after that, the expression of vimentin, *β*-catenin, and survivin was quantified. Apoptosis, cell cycle, and invasion were analyzed by flow cytometry and scratch test, respectively. ZER could significantly sensitize CAFs cells to 5-FU. A combination of 5-FU + ZER revealed a marked decrease in the marker of interest in both mRNA and protein levels compared to control groups, including 5-FU, ZER treated, and untreated cells. Functional evaluation of cells in different groups presented significant suppression in migration of CAFs and an apparent increase in cell arrest and apoptosis by 5-FU + ZER treatment.

## 1. Introduction

Colorectal cancer (CRC) is the most common malignant worldwide. In the past decades, the survival rate of patients with cancer solid tumors has not improved and drug resistance has become one of the important challenges in cancer treatment. All cancers are dependent on different molecular pathways, which can induce diverse mechanisms of resistance to treatment with single agents [1–3]. Additionally, most drug resistance studies have focused on the genetics and epigenetics of cancer cells, while cellular components of the tumor microenvironment (TME) have been ignored [1, 4]. TME is effective in the penetration, distribution, and metabolism of cancer therapeutic agents also plays critical role in cancer chemotherapy resistance [5]. TME is complex and includes nontumor cells that are useful for tumors [6].

One of the most abundant cell types in the TME is cancer-associated–fibroblast (CAFs) [7]. CAFs are involved in the regulation of essential events of cancer such as extracellular matrix formation (ECM), epithelial-mesenchymal transition (EMT), and inflammation; therefore, CAFs have been regarded as a potential therapeutic target for cancer [8, 9]. CAFs are known for the high expression level of smooth muscle-actin (*α*-SMA) and fibroblast-activated protein (FAP). The main markers commonly used for diagnosing CAFs are *α*-SMA and FAP [10]. Also, CD31, E.pCAM, and cytokeratin are negative markers because CAFs do not have epithelial and endothelial properties. It should be noted for tumor eradication, and CAFs must be considered [11, 12].

The epithelial-mesenchymal transition (EMT) process is necessary for tumor metastasis and drug resistance [13]. Research has shown that CAFs play a key role in resistance to chemotherapy by inducing EMT in cancer [14]. EMT is related to different signaling cascades, including the transforming growth factor-*β* (TGF-*β*), Wnt, Hedgehog, and Notch pathways, and it can affect the involved genes such as *β*-catenin which is activated in the Wnt pathway [15]. The Wnt/*β*-catenin cascade is the critical signaling involved in the EMT process of chemotherapy resistance in cancers, particularly in CRC [16, 17]. *β*-Catenin is a part of the protein complex that forms adherens junction [18]. The *β*-catenin is upregulated in different types of cancers [19, 20]. Another gene that plays an important role in EMT is Vimentin, a major component of the intermediate filament family of proteins, which is expressed in mesenchymal cells and is often used as a marker of EMT during both normal growth and metastatic progression of cancer [21, 22]. The expression level of vimentin increases during cancer migration and invasion [23]. Survivin, a protein caspase inhibitor, is the smallest member of the inhibitor of apoptosis (IAP) family [24]. It is associated with resistance to radiotherapy and chemotherapy along with poor prognosis [25]. The inhibition of survivin is considered an attractive strategy for cancer-specific treatment [26]. Because its expression is largely limited to neoplastic tissues, targeting survivin could minimize toxicity to noncancerous cells [27].

Most therapeutic approaches in cancer treatment are associated with toxicity, side effects, lack of selectivity, high cost, and chemoresistance. Therefore, alternative treatment agents are important and the use of herbal components becomes an important option for CRC treatment [28]. 5-FU is commonly used as an effective drug to treat many types of cancers including breast, anal, stomach, head, and neck cancer. However, the response rate of 5-FU for advanced CRC is only 20%, whereas combining 5-FU with other chemotherapeutic agents has increased the response rates up to 40–50% in cancer patients [29].

Recently, novel strategies have been focusing on combining therapy due to increasing the rate of durable clinical responses in cancer patients [30].

Herbs, plants, and plant-based compounds that are commonly referred to as safe compounds have been demonstrated to exert chemopreventive features and mediate anti-cancer roles in the diverse cell. Generally, some natural products including curcumin and ZER are used to treat cancer due to their low toxicity and lesser side effect compared with chemotherapy drugs [31, 32]. Natural drugs exhibit anti-tumor activities through a variety of mechanisms, including downregulation of survival signaling pathways, apoptosis induction, angiogenesis inhibition, and removal of oxidative stress [26, 33].

5-FU is commonly used as an effective drug to treat many types of cancers including breast, anal, stomach, head, and neck cancer. However, the response rate of 5-FU for advanced CRC is only 20%, whereas combining 5-FU with other chemotherapeutic agents has increased the response rates up to 40–50% in cancer patients [29].

ZER is a chemopreventive compound derived from the rhizomes of the wild ginger, *Zingiber zerumbet* Sm.. Recently, anti-tumor effects of ZER have been reported to induce apoptosis and decrease cell proliferation in several cancers, including CRC [34, 35]. ZER has shown its anti-cancer effects by significantly suppressing cell growth and survival [32]. It also reduces angiogenesis, invasion, and metastasis in the cancer cells [36]. ZER could activate different signaling pathways such as FAK/PI3K/NFK*β*-uPA in colorectal cancer [31]. Several types of research have currently demonstrated the therapeutic potential of ZER against various cancers [32]. The present study suggests the synergistic effect of the combination of both ZER and 5-FU in increasing the sensitivity of isolated CAFs to 5-FU treatment. We attempted to demonstrate that ZER treatment via targeting the important genes involved in cancer chemoresistances such as vimentin, survivin, and *β*-catenin could reduce 5-FU resistance in CAFs. Consequently, combination treatment of ZER with 5-FU is expected to prove more beneficial for CRC patients.

## 2. Materials and Methods

### 2.1. Chemical Reagents

ZER (chemical name: (2E, 6E.10E)-2,6,9,9-tetramethylcycloundeca-2,9,10-terien1-one), dimethyl sulfoxide (DMSO), and trypan blue were purchased from Sigma (St. Louis, MO, USA). ZER was dissolved with 1 mL DMSO to make a 229.35 mM stock solution and kept at –20 Cо. The working concentration of ZER was diluted in Dulbecco, s modified Eagle, s medium (DMEM) containing 4% FBS. The 5-FU was purchased from Sigma-Aldrich, and 50 mg of 5-FU was dissolved with 1 mL dH_2_O to make a 384 mM stock solution and kept at room temperature. The future dilution was added in a DMEM medium containing 2% FBS to treated cells. 3-(4,5-dimethylthiazol-2-yl)-2,5 diphenyl tetrazolium bromide (MTT) was prepared from Sigma Aldrich. Primary monoclonal antibodies against *α*-SMA, EPCAM, vimentin, survivin, and GAPDH were purchased from Abcam (Cambridge, AM, USA), and the secondary horseradish peroxidase (HRP)-conjugated, IgG-TR (Texas RED), and IgG FITC were purchased from Santa Cruz Biotechnology, Inc. (Santa Cruze, CA). A high pure RNA isolation kit was obtained from Roche Applied Science (Germany), and SYBR Green QPCR Master Mix was purchased from Thermo Fisher Scientific (USA). The first-strand cDNA synthesis kit was obtained from Thermo Scientific (USA).

### 2.2. Isolation of Fibroblastic-Like Cells

Fibroblast-like cells were isolated from human rectal tumor tissue according to the protocol of similar previous studies [37, 38]. A rectal tumor was obtained from a 67-year-old man from Hamadan province with the symptom of severe diarrhea who was undergoing surgery for the first time. There was no report of drug resistance in the persons' medical record, and this experiment was performed under the suppression of the medical ethics of Hamadan University of Medical Sciences (N0, IR.UMSHA.REC.1399.207) and consent was obtained from the person.

The tumor tissue was first transferred to the laboratory with DMEM medium (Gibco, Invitrogen, USA) containing 20% penicillin/streptomycin/amphotericin B (Invitrogen, USA). Tissues were cut into 2–3 mm^3^ fragments and placed into 50 ml sterile falcon tubes containing 20% penicillin/streptomycin/amphotericin B, followed by 1 h rotation at RT. Afterward, five to eight tissue fragments were placed into 6-well cell culture plates containing 10% gelatin (Gibco, MA) coated in the bed of the well, and medium containing 75% DMEM 20% FBS, and 5% penicillin/streptomycin/amphotericin B at 37°C in a 5% CO_2_-humidified atmosphere. Fibroblastic-like cells and epithelial-like cells were effused from the margin of tissue fragments after approximately 3 weeks, and then tissue fragments were removed. Colonies of fibroblastic-like cells at different palaces of wells were removed by pipet pasture and cells were transferred in 24-well cell culture plates. To homogenize the fibroblastic-like cells and minimize the epithelial-like cells that have entered the well, a serial dilution of cells was prepared with culture medium and each dilution was transferred to wells. Then cells were maintained in appropriate growth conditions until they reached a density of about 70%. The wells with the least contamination of epithelial-like cells were selected for the propagation of fibroblastic-like cells, and seeded first in 6-well cell culture plates and then in 25 cm cell culture flask. Finally, isolated fibroblastic-like cells were cryopreserved in liquid nitrogen.

### 2.3. Characterization of Fibroblastic-Like Cells

To characterize and determine the percentage of isolated cells, we detected a specific CAF marker, *α*-SMA, in fibroblastic-like cells isolated by flow cytometry. The cells were first cultured in a 25 cm cell culture flask and then detached with trypsin-EDTA (1x) and washed twice with cold PBS. Cells were fixed with 3.7% formaldehyde for 10 min and permeabilized with 0.1% Triton-X for 10 min before incubation with mouse anti-human *α*-SMA (ab5694, 1 : 200) antibody (ab5694, 1 : 200). Cells were washed with 0.1% bovine serum albumin (BSA) and then incubated in the anti-mouse IgG FITC conjugate (1 : 50, Santa Cruze) for 1 h. Then, cells were stained and suspended in 0.1% BSA. Negative control of nonfluorescent cells was used to determine the background fluorescence so that less than 1% of nonfluorescent cells were included in the sort gate.

### 2.4. Purification of Fibroblastic-Like Cells by FACS

Separation of fibroblastic-like cells from epithelial-like cells in the cell culture flask was performed by fluorescence-activated cell sorting (BD Biosciences, San Jose, CA, USA) (FACS) using CAF and epithelial-specific markers, *α*-SMA (ab5694, 1 : 200) and E.PCAM (ab323/A3,1 : 200), respectively. After cells reached 70% confluency, they were detached with trypsin 1X and transferred to 15 mL sterile falcon tubes, followed by centrifuging in 400*g* for 5 min. The conditioned media of cells were discarded, and cells were washed twice with 1 ml PBS/PSA. Later, suspension cells were incubated with both specific antibodies for 30 min at RT. Next, 1 ml PBS/BSA was added to cells and centrifuged in 400*g* for 5 min, and finally, 2.5 ml PBS/BSA was added to the cell suspension, and suspended cells were used for sorting by FACS.

### 2.5. Characterization of Purified Cells by Immunocytochemistry (ICC)

After the separation of *α*-SMA positive from EPCAM positive cells, immuno-cytochemical staining was performed to determine *α*-SMA protein in purified fibroblastic-like cells. The cells were first spread on slide glasses and fixed in 4% ice-cold paraformaldehyde plus 0.1% Triton X-100 for 20  min and then blocked with 2% bovine serum albumin in PBS for 1 h. Cells were incubated with anti-*α*-SMA (ab5694, 1 : 200) and then treated with secondary antibody IgG FITC conjugate (1 : 50, Santa Cruze). Finally, coverslips mounted were taken with fluoromount-G, and immunofluorescent images using the OLYMPUS IX71-F22FL/PH microscope (Japan, magnification ×200).

### 2.6. Characterization of Purified Cells by Real-Time PCR

Characterization of purified cells was continued by examining their specific markers in mRNA level. The total RNA was first extracted from cells using the high pure RNA isolation kit according to the manufacturer's protocol. The cDNA was then synthesized from total RNA using RevertAidTM First Strand cDNA synthesis kit following the manufacturer's instruction for incubation at 25°C for 5 min, 42°C for 1 h, and 70°C for 5 min. Next, a quantitative SYBR Green polymerase chain reaction (PCR) was performed. The cycling conditions were: 15 min at 95°C, 30 s at 60°C, and 20 s at 72°C. The specific primer sequences were designed for *α*-SMA, FAP, and 18 srRNA (as internal control). Primer sequences are listed in Table 1. The real-time PCR was run on a Cycler Real-Time PCR light system in replications. The gene expression data for isolated cells were obtained before cell sorting as a control sample.

### 2.7. Cell Viability Assay

MTT assay was used to determine the inhibitory effects of ZER and 5-FU on CAFs viability. CAFs were seeded at a concentration of 1 × 10^4^ cells per well into a 96-well plate and incubated overnight. The next day, the cells were treated with different concentrations of 5-FU (0–300 *μ*M), and ZER (0–100 *μ*M) for 24 h, 48 h, and 72 h. Also, CAFs were simultaneously treated with different concentrations of 5-FU(0–100 *μ*M) and 22 *μ*M of ZER, for 24 h, 48 h, and 72 h. Later, 10 *μ*L of 5 mg/mL MTT reagent was added to each well followed by incubation at 37°C for 4 h. The medium was then discarded and 100 *μ*L of DMSO was added to each well. Finally, the absorbance of solubilized formazan was quantified using an automatic multiwell plate reader(Ryto RT-2100c, China) at 570 nm. The growth inhibitory effects of ZER, 5-FU, and ZER + 5-FU were evaluated by determining IC50 values.

### 2.8. Treatment with ZER and 5-FU

CAFs were treated by combining the ZER (22 *μ*M) and 5-FU (50 *μ*M) for 72 h and, we treated the control group, including 300 *μ*M of 5-FU for 72 h, 22 *μ*M of ZER for 48 h, and untreated CAFs. We also treated 5 × 10^5^ cells/well in 2 ml of media containing 4% FBS for ZER treatment and 2% FBS for 5-FU treatment.

It should be noted that to obtain the best result in each treatment four different IC50 were used for CAFs (19, 20, 22, and 24 *μ*M of ZER and 50, 100, 200, 250, 300 *μ*M of 5-FU) in 24, 48, and, 72 h. Later, the optimum IC50 in the best time treatment was chosen. The combination of both drugs may induce cytotoxicity in cells; therefore, we first treated cells with ZER for 4 h, and after that, the cells were treated with 5-FU over the optimum time (72 h for 5-FU and combination treatment groups). Finally, the conditioned media of cells was removed and replaced by complete media. After 24, 48, and 72 h, cells were trypsinized and used for further analyses.

### 2.9. Real-Time PCR

SYBR green qPCR was performed to evaluate the inhibitory effect of ZER and 5-FU on survivin, vimentin, and *β*-catenin mRNA level. First, the total RNA was extracted from the cells in different groups (5-FU, ZER, 5-FU + ZER treated and untreated). Next, the cDNA was synthesized from total RNA using RevertAidTM First Strand cDNA synthesis kit following the manufacturer's instruction of incubation at 25°C for 5 min, 42°C for 1 h, and 70°C for 5 min. Next, PCR amplification reactions were consequently applied under the following conditions: 15 min at 95°C, 30 s at 60°C, and 20 s at 72°C. The specific primer sequences were designed for survivin, vimentin, *β*-catenin, and 18 srRNA. The real-time-PCR data was analyzed using the∆∆Ct method, and gene expression levels were measured by 2^−∆∆Ct^.

### 2.10. Western Blot Analysis

1 × 10^7^ CAFs cells were trypsinized and washed twice with cold PBS. Then cells were directly lysed in 0.2 ml of ice-cold RIPA buffer (Santa Cruz, USA) and centrifuged at 13,000*g* for 20 min at 4°C. The concentrations of total protein were measured by Bradford assay, and proteins were separated on SDS-PAGE gel (50 *μ*g). Later, protein bands were transferred to nitrocellulose membranes followed by incubation with primary antibodies [Rabbit monoclonal anti-*β*-catenin (ab40772,1 : 7,000), Rabbit monoclonal anti-vimentin (ab24525, 1 : 1000), Rabbit polyclonal anti-survivin (ab469,1 : 700) (anti-rabbit antibody conjugated with HRP)]. Chemiluminescence detection kit (ECL, Amersham, USA) was used to visualized protein bands and further quantified by densitometry, using image j software.

### 2.11. Scratch Assay

Scratch assay was performed to evaluate *in vitro* CAF cell mobility. Cells were seeded in 24-well Trans-well plates at the concentration of 1 × 10^5^ cells/well until they reached 80–90% confluency. Later, cells were treated with ZER, 5-FU, and a combination of both (ZER + 5-FU). Then, the cell monolayers were scratched with 100 *μ*l pipette tips and using PBS, the wells were washed to remove cell debris. To measure and compare the distance of scratches in different groups, the images of cells were captured at different time points (0, 24, 48, 72 h). Quantification of the scratching area was analyzed by the National Institutes of Health (NIH) Image J software.

### 2.12. Colony-Forming Assay

A colony-forming assay was performed to determine the capability of a single cell to grow into a large colony through clonal expansion. To this end, 3 × 10^5^ CAFs Cells were plated in 6-well Trans-well plates and incubated overnight. Later, cells were treated with ZER, 5-FU, and a combination of both (ZER + 5-FU) for 72 h, and then the viable cells were then collected. CAF cells (3,000) were seeded into a 100 mm dish and were subsequently incubated for 7–10 days at 37°C in a humidified 5% CO_2_ atmosphere. To evaluate the survival of cells in different groups, all the colonies were stained with crystal violet (2%).

### 2.13. Cell Cycle Analysis

CAF cells treated in different groups were fixed overnight with 70% ethanol in ice-cold PBS at –20°C, then resuspended with propidium iodide (PI) (Sigm*α*-Alrich; Merck KgaA, Darmstadt, Germany) and RNase A (Sangon Biotech Co., Ltd., Shanghai, China) and incubated in the dark for 15 min at RT. The DNA contents of samples were analyzed using a FACS Calibur flow cytometer (BD Biosciences, San Jose, CA, USA).

### 2.14. Annexin V/PI Staining

The apoptotic cell number was evaluated using flow cytometry. Control and treated cells were harvested, washed twice with cold PBS, and stained with the Annexin V and propidium iodide (PI). The cells were incubated for 15 min in dark conditions. The percentage of apoptotic cells was measured by flow cytometry using the Cell-Quest software.

### 2.15. Statistical Analysis

Data analysis was done using the SPSS16.0 software. All data were presented as mean ± S.E.M. Data were compared by analysis of variance (one-way ANOVA). A *p* value less than 0.05 was considered statistically significant.

## 3. Results

### 3.1. Isolation and Characterization of Fibroblastic-Like Cells

**Figure 1:**
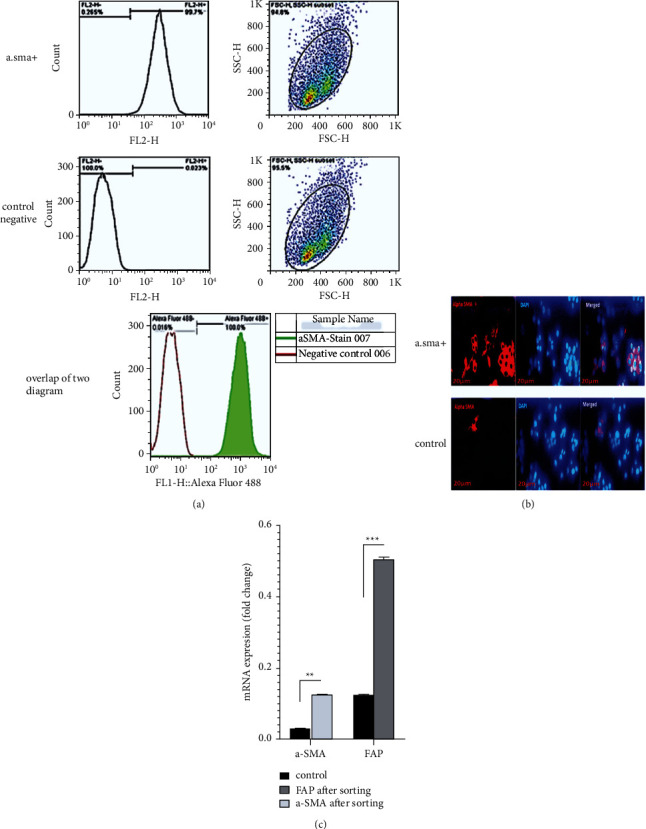
Isolation and characterization of fibroblastic-like cells from human CRC tumor. (a) Characterization of fibroblasts: primary human fibroblast isolated from tumor was characterized by flow cytometry for *α*-SMA marker (green). *α*SMA-positive fibroblastic cells were upregulated in cells after sorting compared to control. (b) The level of *α*-SMA expression in fibroblastic cells analyzed by ICC, *α*-SMA positive cells (fibroblastic cells after FACS), and control cells (fibroblastic cells before FACS). (c) FAP and *α*-SMA mRNA level measured by real-time PCR (relative to before sorting as a control). Mean ± SEM. ^*∗∗*^*p* < 0.05, ^*∗∗*^*p* < 0.001compared to control (unsorted control).

The isolated cells exhibited typical fibroblast characteristics: spindle shape, large swollen that line up in a line, and polygonal. Isolation of fibroblast-like cells from tumor tissue was performed. Initially, fibroblastic-like cells protrude from the edge of the tumor tissue fragments. The fibroblast cells migrated and spread into the well and formed cell clones. A cell clone was removed, purified, and propagated in cell culture flasks. We performed FACS to isolate *a*-SMA-positive cells from EPCM-positive cells. Results presented that 99.7% of purified cells were *α*-SMA positive (Figure 1(a)). Further FACS-sorted fibroblastic-like cells were confirmed by immunohistochemical staining for *α*-SMA markers. ICC results demonstrated positive staining for *α*-SMA intracytoplasmic fibroblastic-like cells. ICC detected *α*-SMA positive cells (73%) and the presence of CAF cells was confirmed once more (Figure 1(b)). Moreover, we carried out the Real-time-PCR to evaluate the expression level of *α*-SMA and FAP in purified fibroblast-like cells. Results showed that mRNA levels of FAP and *α*-SMA in purified fibroblastic-like cells were significantly increased by 29% and 64%, respectively, compared to before purification by FACS shown in (Figure 1(c)).

### 3.2. ZER Intensifies Suppression of CAF Cells Viability

**Figure 2:**
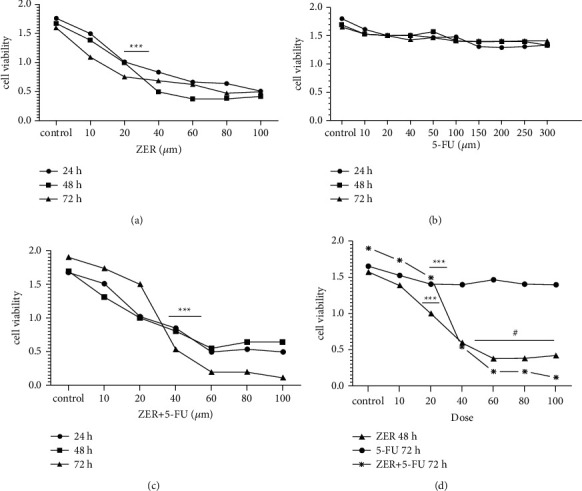
The effect of ZER on CAF cells viability. (a) Various concentrations of ZER (0–100 *μ*M) and (b) 5-FU (0–300 *μ*M), and (c) combination of both for 24 h, 48 h, and 72 h. (d) Mix of three treatments on the proliferation of CAFs were determined using MTT assay. Inhibitory concentration (IC50) of ZER, and ZER + 5-FU (22 *μ*M and 50 *μ*M, respectively). No inhibition of growth was seen by 5-FU alone. Data are reported as the mean ± SME (=3). ^*∗∗∗*^*p* < 0.0001, ≠*p* < 0.05 compared to control (untreated) and ZER treated, respectively. Each data point is presented as mean ± SED (*n* = 3).

We determined the effect of ZER and 5-FU on CAF cells viability by MTT assay. The cells were treated with several doses of ZER and 5-FU for 24, 48, and 72 h. As shown in Figures 2(a)and 2(b), ZER reduced the percentage of viable cells compared to untreated cells in a dose and time‐dependent manner. The IC50 for ZER was detected at 22 *μ*M, while no inhibitory effect was observed by 5-FU. We also found that in the treatment of cells with both ZER and 5-FU, the amount of IC50 was decreased for 5-FU and cell growth inhibition was observed in concentration of 50 *μ*M of 5-FU (Figure 2(c)). Therefore, concentrations of 22 *μ*M and 50 *μ*M of ZER, and 5-FU were selected for the further experiment, respectively.

### 3.3. ZER Reinforces Suppression of CAF Cell Proliferation

**Figure 3:**
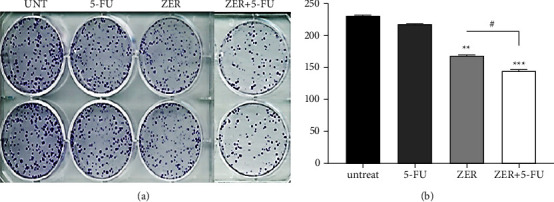
The effect of ZER on colony-forming of CAF cells. Cells were treated with ZER (22 *μ*M), 5-FU (300 *μ*M), and ZER + 5-FU (22 *μ*M and 50 *μ*M, respectively) for 48 h. Significant inhibition was found in ZER treated group. A more inhibitory effect was observed by the ZER + 5-FU treatment. Each data point is presented as mean ± SED (*n* = 3). ^*∗∗*^*p* < 0.01, ^*∗∗∗*^*p* < 0.001 compared to control (untreated), ^#^*p* < 0.05 compared to ZER treated group.

Here, we presented that CAF colony-formation cells were significantly suppressed by ZER, and 5-FU presented no inhibition on cell proliferation. Besides, treatment of cells with ZER + 5-FU defined more significant inhibition of colonies through the potentiating effect of the ZER on 5-FU (shown in Figures 3(a) and 3(b)).

### 3.4. ZER Increases CAF Cells Apoptosis

**Figure 4:**
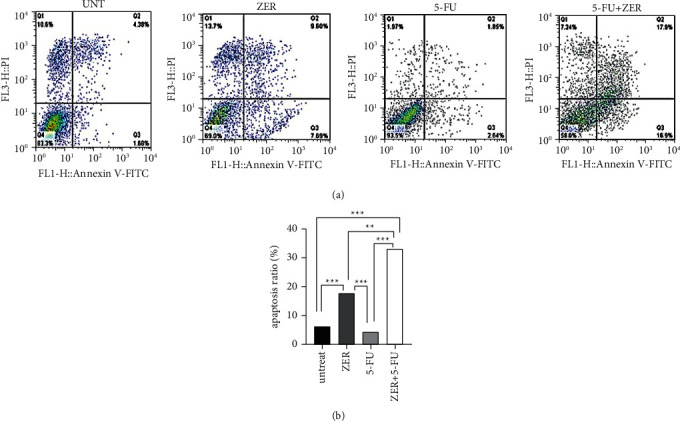
The effect of ZER on apoptosis of CAF cells. (a) CAF cells were stained with AnnexinV-PI after treatment with 22 *μ*M of ZER for 48 h and 50 *μ*M of 5-FU for 72 h, and ZER + 5-FU for 72 h. (b) The ZER and ZER + 5-FU could promote cell apoptosis vs. the untreated and 5-FU treated cells, respectively (^*∗∗∗*^*p* < 0.001^*∗∗∗*^*P* < 0.001) and (^*∗∗*^*p* < 0.01^*∗∗*^*P* < 0.01) (*n* = 1).

We presented that ZER considerably increased cell apoptosis in 48 h (^*∗∗∗*^*p* < 0.001^*∗∗∗*^*P* < 0.001). There was no significant increase in the 5-FU-treated group (Figure 4). Treatment of cells with ZER + 5-FU presented a considerable increase in apoptosis for 72 h compared to any single treatment (^*∗∗*^*p* < 0.001^*∗∗*^*P* < 0.001) (Figure 4(b)).

### 3.5. ZER Improves Cell Cycle Arrest

**Figure 5:**
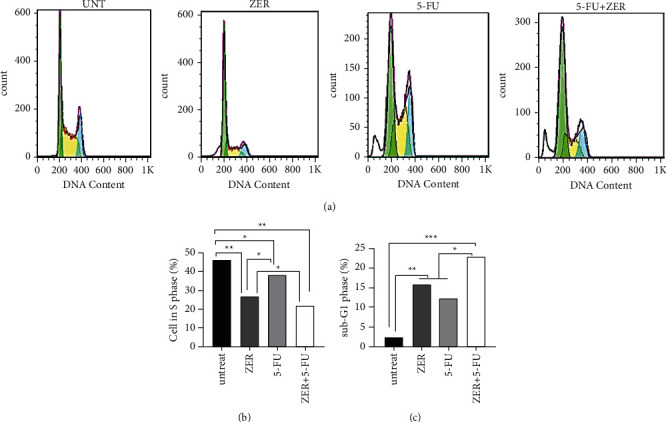
The effect of ZER on the cell cycle of CAF cells. (a) CAF cells were incubated with propylene sodium after treatment of CAF cells with 22 *μ*M of ZER for 48 h and 50 *μ*M of 5-FU for 72 h. (b) CAF cell cycle was arrested in S phase markedly in ZER, and ZER + 5-FU treated cells vs. the untreated and 5-FU treated, respectively (^*∗∗*^*p* < 0.01^*∗∗*^*P* < 0.01) (^*∗*^*p* < 0.05^*∗*^*P* < 0.05). (c) The ZER and ZER + 5-FU could increase apoptotic cells in sub-G1 vs. the untreated and 5-FU treated cells, respectively (^*∗∗*^*p* < 0.01^*∗∗*^*P* < 0.01) (^*∗*^*p* < 0.05^*∗*^*P* < 0.05) (*n* = 1).

We determined a significant cell cycle arrest in the S phase in CAF cells treated with ZER for 48 h compared to untreated cells (^*∗∗*^*p* < 0.01^*∗∗*^*P* < 0.01) (Figure 5(a)). There was no significant suppressing effect in 5-FU-treated cells. Besides, the combination of both ZER and 5-FU markedly elevated S phase arrest in CAF cells for 72 h compared to the untreated group (^*∗∗*^*p* < 0.01^*∗∗*^*P* < 0.01) and ZER-treated group (^*∗*^*p* < 0.05) (Figure 5(b)). Also, ZER, 5-FU, and combination of both ZER and 5-FU markedly increased the sub-G1 phase percentage cells in CAF cells for 72 h compared to the untreated group respectively (^*∗*^*p* < 0.05) (^*∗∗*^*p* < 0.01^*∗∗*^*P* < 0.01), and ZER-treated group (^*∗*^*p* < 0.05^*∗*^*P* < 0.05).

### 3.6. ZER Intensifies Downregulation of *β*-Catenin, Survivin, and Vimentin in mRNA and Protein Level

**Figure 6:**
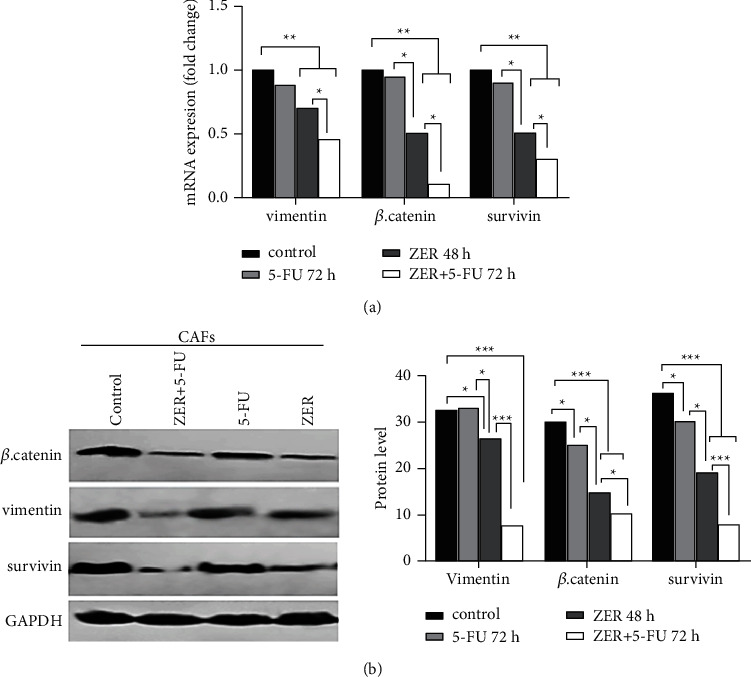
The effect of ZER on the expression of *β*. Catenin, survivin, and vimentin by real-time-PCR and Western blotting. CAF cells were treated with 22 *μ*M ZER for 48 h, 50 *μ*M 5-FU for 72 h, and 5-FU + ZER for 72 h. (a) ZER and ZER + 5-FU could markedly inhibit all three biomarkers in mRNA and protein level compared to untreated and ZER-treated groups, respectively (^*∗∗*^*p* < 0.01^*∗∗*^*P* < 0.01 vs untreated) (^*∗*^*p* < 0.05^*∗*^*P* < 0.05 vs ZER-treated) in mRNA level, and (^*∗∗∗*^*p* < 0.001 vs untreated), and (^*∗*^*p* < 0.05^*∗*^*P* < 0.05, ^*∗∗∗*^*p* < 0.001^*∗∗∗*^*P* < 0.001 vs ZER treated). Each data point is presented as mean ± SED (*n* = 3).

We demonstrated that ZER dramatically inhibits mRNA level of all three invasive biomarkers.

(^*∗∗*^*p* < 0.01^*∗∗*^*P* < 0.01) 5-FU could not significantly decrease their mRNA levels compared to untreated cells. Besides, marked inhibition of each biomarker in mRNA level was observed in CAF cells treated with ZER + 5-FU compared to treatment alone (^*∗*^*p* < 0.05^*∗*^*P* < 0.05) (Figure 6(a)). The changes in mRNA level of the biomarkers of interest were confirmed by Western blotting, and the same findings were found in the protein level of *β*. catenin, survivin, and vimentin (Figure 6(b)).

### 3.7. ZER Increases Suppression of CAF Cells Migration

**Figure 7:**
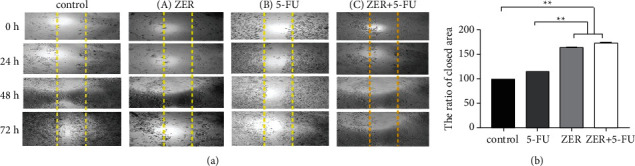
The effect of ZER and 5-FU on CAFs cell migration. (a) CAFs were treated with 5-FU, ZER, and ZER + 5-FU at different time points. (b) Semiquantitative analysis of migration assay, significant reduction of cell migration in ZER, and combination of ZER + 5-FU.

We evaluated the effect of ZER and 5-FU on the migration of CAFs. The results showed that 5-FU had no inhibitory effect on CAF cell migration. Moreover, treatment of cells with ZER and ZER + 5-FU significantly suppressed CAF cell migration compared to untreated cells and the 5-FU-treated group (^*∗∗*^*p* < 0.01^*∗∗*^*P* < 0.01) (Figure 7).

## 4. Discussion

In recent years, despite significant advances in screening, diagnosis, and new treatment, the survival rate of people with solid cancers has not changed significantly [39]. Drug resistance in cancers is considered one of the main reasons [1]. Recently, many research studies have focused on the anti-cancer effects of natural products and indicated that these agents are suitable alternatives for inhibiting numerous cancers. One of these plant derivatives is ZER, extracted from the plant's root from the ginger family called *Zingiber zerumbet* Sm. [40]. Many research indicated that ZER reduces cell migration and proliferation and induces apoptosis in different cancers [41]. The valuable effects of ZER on prostate and nonsmall cell lung carcinoma cancer have also been identified in chemotherapy and radiotherapy resistance [33, 42].

So far most studies about drug resistance have been performed on the genetics and epigenetics of cancer, while the TME plays an essential role in creating drug resistance [43]. Smith et al. found that CAFs induced head and neck cancer cell resistance by activating TGF-*β*, Snail, and Twist [44]. Another study has reported that exosomes secreted by CAFs can activate the Notch pathway, which causes chemotherapy resistance in breast cancer [45]. Another study presented that activation of the Wnt/*β*-catenin signaling is mediated by CAF exosomes resulting in CRC cell's resistance to 5-FU [46].

Here, we demonstrated the inhibitory effect of ZER on CAF cells which could improve the response of CAF cells to 5-FU. For this reason, we first isolated fibroblast-like cells from human colon cancer tissue and characterized CAF cells by demonstrating CAF-related markers, including *α*-SMA and FAP, by flow cytometry, ICC, and real-time-PCR in isolated cells.

MTT assay revealed that ZER significantly decreased cell proliferation and sanitized CAF cells' response to 5-FU in a time-and dose-dependent manner (^*∗∗∗*^*p* < 0.0001). From MTT results, we found that the cells of our interest are not responsive to 5-FU up to a dose of 300 *μ*M when treating CAF cells with 5-FU and ZER, the IC50 value of 5-FU was dropped to 50 *μ*M to suppress CAF cells growth. This finding was interesting and presented the powerful effect of ZER on CAF cells that sensitizes the cells to 5-FU.

We then introduced the potentiating effect of ZER on 5-FU, which could result in downregulation of the targets of our interest, including vimentin, survivin, and *β*-catenin. ZER significantly reduced expression of all three biomarkers in mRNA levels (30%, 50%, 50%) and protein levels (19%, 47%, 51%), respectively, alone. It should be noted that in the group of cells with combination treatment, ZER can sensitize cells into 5-FU. The biomarkers were significantly reduced compared to the ZER-treated cell alone in mRNA levels (55%, 70%, 90%) and protein levels (80%, 78%, 67%), respectively. Vimentin is a mesenchymal marker of the EMT process that its overexpression is associated with many cancers [44]. The *β*-catenin is a key driver of Wnt/*β*-catenin signaling. Survivin is an important mediator of tumor growth, invasion, and metastasis through its anti-apoptotic function [47, 48]. In agreement with our result, Dermani et al. demonstrated ZER repressed the EMT process by inactivating Wnt/*β*-catenin signaling. The protein expression levels of N cadherin, vimentin, ZEB1, nuclear *β*-catenin, c-Myc were also suppressed by ZER in a concentration-dependent manner [49]. Another study demonstrated that cotreatment of 5-FU and resveratrol can inhibit the EMT pathway and down-regulate vimentin expression in colorectal cancer cells [50]. Pandey et al. have observed that the combination of 5-FU with herbal components included Curcumin “berberine” and quercetin sensitize cells to 5-FU and decrease expression of survivin and STAT3 in gastric cancer cells [51]. Regarding colony-formation assay, we found significant inhibition in ZER-treated group and the more inhibitory effect was observed in the combination ZER + 5-FU treatment group compared to the untreated (^*∗∗*^*p* < 0.01) and, ZER-treated group (^#^*p* < 0.05). Here, based on the results obtained from isolation procedures, it can be assumed that the results are mostly related to CAF cells and the percentage of cancer cells that may affect the results is not considerable. Regarding the cell cycle analysis, we reported that ZER induced cell cycle arrest in the G1 phase (32.73%), while 5-FU did not change cell cycle; when it comes to cells treatment with a combination of both, reduction of the cell cycle in G1 was significant compared to ZER-treated group (52.95%). These findings are in agreement with other studies, such as ZER-induced arrest cell cycle at the G2/M phase in ovarian cancer [52]. ZER-induced cell cycle arrest and apoptosis in oral squamous cell carcinoma (OSCC) by activating the Akt and S6 proteins and inhibiting the PI3K-mTOR pathway in a dose-time-dependent manner [53]. ZER by increasing p27, cytochrome c, caspase-3 caspase-9, and decreasing cyclin-dependent kinase 1, in mRNA and protein level, arrested the cell cycle at the G2/M phase, thereby suppressing the invasion of HepG2 cells [54]. Another study reported that ZER enhances cell cycle arrest in the G2/M phase and increases apoptosis through upregulation of the Bax/Bcl-2 ratio of human GBM U-87 MG cells [55].

The predisposition of cells in the cellular apoptosis was investigated, and it was found that ZER could significantly increase the apoptosis of CAF cells alone (17.59%). 5-FU did not upregulate cellular apoptosis in CAF cells, but in combination with ZER, apoptosis increased significantly (33%). Similar to our study combination of 5-FU, and Rutin (a glycoside from quercetin flavonoid) increases the expression of *P53*, *Bax*, and *Bak* genes and induces apoptosis in prostate cancer [56]. Another study reported that combining 5-FU and resveratrol increases apoptosis in HTC116 and DLD1 cells by inhibiting AKT and STAT3 signaling [57].

Liu et al. suggested that cotreatment of 5-FU and *Sanguisorba officinalis* L. radix (DiYi) may enhance apoptosis of colorectal cells (HTC116 and RKO) through activating oxygen species, mitochondrial-caspases-dependent apoptosis pathway more than employing each alone [58]. It has been reported that cotreatment of 5-FU with ethanol extract of *Spica prunellae* (EESP) can increase apoptosis ratio in HCT-8/5-FU cells compared to treatment with each alone [59]. Additionally, we performed a scratch assay to identify the inhibitory effect of ZER and 5-FU on the migration of CAF cells. Based on our findings, no inhibition happened by 5-FU. At the same time, it was significant by ZER (^*∗∗∗*^*p* < 0.001). Besides, the combination of ZER and 5-FU markedly suppressed (5.5%) migration of CAF cells compared to ZER-treated cells. In agreement with our results, Hosseini et al. reported that ZER could reduce migration and metastasis in colorectal cancer by inhibiting FAk/PI3k/NF-*κ*B-uPA signaling pathway [31]. Another study has suggested that ZER reduces cell migration in esophageal squamous cell carcinoma (ESCC) cells by ubiquitination of Roc1 [60]. Suppression of nonsmall cell lung cancer (NSCLC) cell migration and invasion has been reported by Hu et al. [61]. It has been reported that a combination of 5-FU with fucosterol inhibits cell proliferation, clonogenic potential, and cell migration without producing cell death in two human CRC cell lines (HCT116 and HT29). Besides, 5-FU showed no inhibitory function in cell migration [62]. The present study results provided valuable insights into the potentiating effect of ZER on 5-FU function in the regulation of the tumor-promoting role of CAF cells.

## 5. Conclusion

In this study, we demonstrated that ZER could significantly diminish proliferation, apoptosis, and migration of CAF cells through suppression of the critical drivers of cancer metastasis and progression. Besides, we presented that ZER could improve the response of CAF cells to 5-FU, showing a potentiating effect of ZER on 5-FU. Our findings may enlighten researchers to consider the ZER-based combination therapy, which can effectively improve the clinical applications of 5-FU against cancers, including colorectal cancer.

## Figures and Tables

**Table 1:** Primer sequences for real-time PCR.

Gene	Forward primer	Reverse primer	Product length (bp)
18 srRNA	GTAACCCGTTGAACCCCAT	CCATCCAATCGGTAGTAGCG	151
*α*-SMA	CACTGCCGCATCCTCATC	TGCTGTTGTAGGTGGTTTCAT	161
FAP	GAAAGAAAGGTGCCAATA	GATCAGTGCGTCCATCA	116
*β*-Catenin	CTTCACCTGACAGATCCAAGTC	CCTTCCATCCCTTCCTGTTTAG	98
Vimentin	CATTGAGATTGCCACCTAC	CGTTGATAACCTGTCCATC	188
Survivin	TGGGAAGGGTTGTGAATGAG	GCTGTCTCTACTTTCCAGGATG	99

## Data Availability

The data used to support the findings of this study are included in the article.
